# Enhancing Public Health Surveillance: Outbreak Detection Algorithms Deployed for Syndromic Surveillance During Arbaeenia Mass Gatherings in Iraq

**DOI:** 10.7759/cureus.60134

**Published:** 2024-05-12

**Authors:** Mustafa Suraifi, Ali Delpisheh, Manoochehr Karami, Yadollah Mehrabi, Katayoun Jahangiri, Faris Lami

**Affiliations:** 1 Department of Epidemiology, School of Public Health and Safety, Shahid Beheshti University of Medical Sciences, Tehran, IRN; 2 Department of Epidemiology, Safety Promotion and Injury Prevention Research Center, School of Public Health and Safety, Shahid Beheshti University of Medical Sciences, Tehran, IRN; 3 Department of Health in Disaster and Emergencies, School of Public Health and Safety, Shahid Beheshti University of Medical Sciences, Tehran, IRN; 4 Department of Community and Family Medicine, College of Medicine, Baghdad University, Baghdad, IRQ

**Keywords:** iraq, mass gatherings, syndromes, outbreak detection algorithms, public health surveillance

## Abstract

Background: Large gatherings often involve extended and intimate contact among individuals, creating environments conducive to the spread of infectious diseases. Despite this, there is limited research utilizing outbreak detection algorithms to analyze real syndrome data from such events. This study sought to address this gap by examining the implementation and efficacy of outbreak detection algorithms for syndromic surveillance during mass gatherings in Iraq.

Methods: For the study, 10 data collectors conducted field data collection over 10 days from August 25, 2023, to September 3, 2023. Data were gathered from 10 healthcare clinics situated along Ya Hussein Road, a major route from Najaf to Karbala in Iraq. Various outbreak detection algorithms, such as moving average, cumulative sum, and exponentially weighted moving average, were applied to analyze the reported syndromes.

Results: During the 10 days from August 25, 2023, to September 3, 2023, 12202 pilgrims visited 10 health clinics along a route in Iraq. Most pilgrims were between 20 and 59 years old (77.4%, n=9444), with more than half being foreigners (58.1%, n=7092). Among the pilgrims, 40.5% (n=4938) exhibited syndromes, with influenza-like illness (ILI) being the most common (48.8%, n=2411). Other prevalent syndromes included food poisoning (21.2%, n=1048), heatstroke (17.7%, n=875), febrile rash (9.0%, n=446), and gastroenteritis (3.2%, n=158). The cumulative sum (CUSUM) algorithm was more effective than exponentially weighted moving average (EWMA) and moving average (MA) algorithms for detecting small shifts.

Conclusion: Effective public health surveillance systems are crucial during mass gatherings to swiftly identify and address emerging health risks. Utilizing advanced algorithms and real-time data analysis can empower authorities to improve their readiness and response capacity, thereby ensuring the protection of public health during these gatherings.

## Introduction

Mass gatherings (MGs) are currently regularly held all over the world. The World Health Organization (WHO) defines an MG as an occurrence in which the participants' medical needs place a burden on the resources at hand and the efficient provision of healthcare services [1]. Every year, religious and secular events, including festivals and sports-related gatherings, occur nationally and internationally. The public health challenges associated with mass gatherings have become increasingly complex nationally and globally [2]. Potential health security risks, such as infectious diseases, outbreaks, noncommunicable diseases, and injuries, have prompted extensive research in this area [3].

The use of outbreak detection algorithms has become increasingly popular in public health surveillance systems globally in the past few years. These algorithms, which typically rely on statistical modeling techniques, allow for the early identification of anomalies in health data, thereby enabling swift response and containment measures. By analyzing various data sources, such as clinical records, syndromic surveillance systems, and real-time data, these algorithms can detect patterns that may indicate potential outbreaks or unusual health events with greater precision and accuracy than traditional surveillance methods alone [4].

One of the world's biggest religious mass gatherings takes place in Iraq. Every year, a vast number of people travel to Karbala, Iraq, to honor the memory of the great Muslim leader, Imam Hussain ibn Ali, on his death anniversary. Millions of people gathered for this historic occasion, known as Arbaeenia, in honor of Imam Hussain. Attendance has increased consistently over time, from 3 million in 2003 and 2008 to 16.327 million in 2021 [5]. In the most recent years, the number of visitors reached 21.198 and 22.019 million in 2022 and 2023 respectively [6].

The Arbaeenia mass gatherings in Iraq present significant challenges to public health surveillance due to the immense influx of pilgrims, creating a heightened risk of disease transmission and health emergencies. Traditional surveillance methods often struggle to cope with the scale and complexity of these events, leading to delays in detecting and responding to potential health threats. As a result, there is a pressing need for innovative approaches to enhance syndromic surveillance and improve the early detection of outbreaks and unusual health events during the commemorations of Arbaeenia [7].

The deployment of outbreak detection algorithms for syndromic surveillance during the Arbaeenia mass gatherings in Iraq represents a proactive approach to addressing the formidable challenges posed by large-scale events in public health surveillance [8]. By leveraging advanced technological solutions, such as statistical modeling, public health authorities aim to enhance their capacity to promptly detect and respond to potential health threats. However, successfully implementing these algorithms requires careful consideration of various factors, including data integration, algorithm refinement, and stakeholder collaboration. Through a comprehensive analysis of the rationale, strategies, and implications of deploying outbreak detection algorithms in the context of Arbaeenia mass gatherings, this article seeks to describe the implementation and effectiveness of outbreak detection algorithms for syndromic surveillance during mass gatherings in Iraq as well as to define the levels of alarm thresholds specific to the studied syndrome.

The importance of this study lies in the critical need to safeguard the public health and well-being of the millions of pilgrims who participate in the Arbaeenia mass gatherings in Iraq. These gatherings pose unique challenges regarding disease surveillance, outbreak detection, and rapid response due to the sheer scale of the event and the potential for the spread of infectious diseases. A syndromic surveillance system tailored to these mass gatherings makes it possible to enhance public health preparedness, mitigate health risks, and ensure the safety of participants and the surrounding communities [9].

## Materials and methods

Study design

We adopted a cross-sectional survey to assess the existing situation, assess the health-seeking behaviors of pilgrims, and assess the most common health-related events (syndromes) including influenza-like illness (ILI), gastroenteritis, food poisoning, heatstroke, and febrile rash syndromes during mass gatherings in Arbaeenia.

Study setting and study population

The present study was conducted along the main road from Najaf to Karbala (Ya Hussain Road). These two governorates are situated approximately 160 km and 100 km southeast of the capital city, Baghdad, respectively. Pilgrims who suffered from certain signs and symptoms (syndromes) and sought medical care through health clinics along the road from Najaf to Karbala over 10 days were included.

Sampling technique and data collection

Our sampling method utilized a systematic random sampling approach to select health clinics located along the road from Najaf to Karbala. Firstly, we determined our desired sample size of 10 health clinics. To achieve this, we divided the total number of health clinics (52) by our target sample size, resulting in an estimated interval between the sequence of health clinics.

Next, we randomly selected a number from 1 to the interval size and chose the first health clinic accordingly. Subsequently, we employed systematic random sampling by following the list of public health clinics and selecting every nth clinic, where n is the determined interval. This approach ensured that all public health clinics along the road were given an equal chance of being included in the sample, minimizing bias compared to a simple random sampling method that might have resulted in selecting clinics primarily at the beginning or end of the road from Najaf to Karbala.

A convenient sample targeted all Arbaeen pilgrims who seek medical care and visit the public health clinics along the major route from Najaf to Karbala over 10 days were first examined by the medical unit physician to determine whether s/he suffered from an acute condition or symptoms (syndromes) related to the communicable disease. Afterward, they were interviewed by the data collector on the prepared information sheet (see Appendices) that was developed for this study. This implies that pilgrims who did not seek medical care within the allotted time frame for data collection (10 days) or who did not exhibit any of the symptoms and indicators included in this study would not be eligible to participate in the study. Ten data collectors participated (from August 25, 2023, to September 3, 2023), cooperating with the Najaf Health Directorate and with technical support from three monitoring supervisors. Because the survey was so straightforward, each case interview lasted anywhere from 10 to 15 minutes. Still, the interviews lasted an average of about 13 minutes.

Training of Data Collectors

After coordination with the Field Epidemiological Training Program (FETP) section in the Public Health Department, a session of training of data collectors, 10 employees were selected from various health institutions, and the principles of experience and previous training in public health programs and surveillance systems were adopted.

Case Definitions

The definitions of priority cases for health indicators that were measured during the Arbaeenia mass gatherings are as follows.

Influenza-like illness (ILI): Visitors who had a measured fever of ≥ 38°C, cough, and sometimes sore throat with onset within the last 10 days [10].

Gastroenteritis: Persons with fever ≥ 38°C and acute bloody or watery diarrhea [11].

Food poisoning: Visitors who had at least one of the most common symptoms of food poisoning include diarrhea, abdominal pain, nausea, vomiting, and fever [12].

Heat stroke: Pilgrims who have confusion, loss of consciousness (coma), hot, dry skin or profuse sweating, high body temperature, headache, and nausea [13].

Febrile rash: Any person with fever, generalized maculopapular, skin rash, or cough [14].

Survey Tool

The KoBoToolbox platform (Cambridge, MA) was used to collect data on a tablet or mobile device via the survey tool. The official surveillance activity (field data entry) was from 8:00 a.m. on August 25, 2023, until 12:00 a.m. the next day, each day for 10 days. First, there were some tests and retests for all the data collectors before starting in the fields and those test entries were removed from the analysis. Data of 12202 pilgrims to formal site-monitored field clinics treating patients who participated in the Arba’een mass gatherings were recorded via real-time surveillance. The case form included all the information mentioned in the prepared information sheet (see Appendices), 149 of the pilgrims who did not consent to participate in the current study for various reasons were listed in the database, and 4938 of the total number of visits we registered were pilgrims who had a syndrome.

Data analysis 

To detect possible outbreaks during the study period and alarm thresholds for events in the syndromic surveillance system, the following statistical methods were used. 

Moving Average Algorithm

The model uses several new actual event datasets to generate forecast values for future events. The moving average method will be effectively applied. Historical data are needed for a certain period to obtain forecast results for the coming period. For example, with the 5 points of the time moving average method, the 6th point of the time forecast can only be calculated after the 5th point of time ends. Additionally, we can represent the point of time for the current study as four hours for each point of time. A known or trustworthy estimate of the standard deviation and the specification of a target value are prerequisites for the use of the moving average chart. Because of this, it is best to use the moving average chart after process control has been established [15].

MA=Z_i_=X_i_+X_i-1_+X_i-2_ ... +X_i-N+1_/N

where Xi is the sample value for time interval i, and N is the length of the moving average.

Shorter periods, such as 5 or 7 time points, result in more sensitive moving averages. They react quickly to recent changes in the data, making them suitable for identifying short-term trends or capturing rapid fluctuations. However, this sensitivity may also lead to more noise in the analysis [15].

Longer periods, on the other hand, result in smoother moving averages. Averaging over a longer period reduces the impact of short-term fluctuations, providing a clearer picture of longer-term trends or patterns. This can help filter out noise and highlight more significant movements [15].

Cumulative Sum (CUSUM) Algorithm

Control charts are quicker, which is one of the known algorithms for the early detection of outbreaks that can identify slight changes in the number of syndromes [16]. CUSUM is under the umbrella of statistical process control-based methods [17], and it is used especially when historical data are not available [18]. The CUSUM procedure has become a standard tool for process control and is the recommended method for the timely detection of small step changes. CUSUM is an efficient way to identify small shifts to an out-of-control state, like the beginning or development of syndromes, by reducing the expected time until a change is signaled once the process has shifted to an out-of-control state [18].

The superiority of the cumulative sum chart approach over scan statistics was demonstrated by Woodall et al. [19]. CUSUM, exponentially weighted moving average (EWMA), and scanned statistics were compared for surveillance data that followed Poisson distributions by Han et al. Their findings demonstrated that the exponentially weighted moving average and cumulative sum charts performed better than the scan statistic method [20].

The CUSUM equation can be written as follows:

CUSUM_t_= MAX (0, CUSUM_t-1_+Y_t_-σ/2)

where Y_t _is the number of reported syndromes at time point t (t = 1, 2... n), CUSUM_t-1 _is the CUSUM value on day t-1, and σ is the standard deviation of the observed data at time points of interest [21].

The upper control limit or alarm threshold level for the CUSUSM algorithm was calculated using a limited period based on a one-sided positive CUSUM calculation. The corresponding formula is shown in equation:

Upper Control Limit = UCL = µ+ h ×σ

where μ is the mean of the observed data at the time of interest, h is an appropriate value (fixed parameter) ranging from 1 to 3, and σ is the standard deviation [21].

Exponentially Weighted Moving Average (EWMA) Algorithm

Epidemic detection techniques are typically included in temporal and spatial approaches as the main tools for syndromic surveillance systems [22]. One of the most well-known methods or algorithms used by syndromic surveillance systems to spot outbreaks or any change in the syndrome trend is the EWMA [22]. A statistical process control chart called the exponentially weighted moving average algorithm is useful for identifying subtle but enduring changes in the syndrome trend. However, unlike EWMA, no single algorithm with fixed parameters can cover a broad spectrum of outbreaks under many conditions and in many environments. Using actual data testing to assess the effectiveness of outbreak detection techniques offers the highest level of validity [23].

These are trend-based alerts based on data. CUSUM is comparable to another statistical quality control method. The EWMA algorithm, as its name implies, is a moving average algorithm variation in which the weights of the current observations are assigned. The EWMA algorithm is used in trend-based alerting to determine average values and to spot anomalous spikes and shifts in the log trend.

The following recursive equation defines the exponentially weighted moving average statistics:

EWMA_(t)_=λ*X_(t)_+(1-λ)*EWMA_(t-1)_

where EWMA_(t)_ is the exponentially weighted moving average at time t.

λ is the smoothing parameter, often ranging between 0 and 1. The closer λ is to 0.0, the greater the effect of previous values averaged into the current value. Conversely, the closer λ is to 1.0, the smaller the smoothing effect. The literature recommends that λ be within the range of 0.05 to 0.3.

X_(t)_ is the current actual value at time t [24].

The control limits and central line of the exponentially weighted moving average (EWMA) chart are calculated using the formula below, where µ0 is the centerline [25].

UCL, LCL=μ0±Lσsqrt{(λ/2-λ)1- [1-λ]^{2t}}

The value μ0 is the process mean. The value σ is the process standard deviation. The constant λ is the user-specified smoothing value (usually in the range 0.05 to 0.30). The width of the control limits is controlled by the constant L, where the typical value for L is 2.7 to 3.0.

The EWMA chart was created in this manner to identify possible "alarm" signs, or stronger outlier signals. Any single observation that deviates from the control ranges indicated by the exponentially weighted moving average control chart is considered an outlier in this context.

Quantitative data were analyzed using the software Statistical Package for Social Sciences (SPSS), version 26.0 (IBM Corp., Armonk, NY), MINITAB statistical software version 21 (Minitab Inc., State College, PA), and Microsoft Excel (Microsoft Corporation, Redmond, WA). The data are presented as numbers and percentages.

## Results

During the study period from August 25, 2023, to September 3, 2023, a total of 12202 pilgrims participated in the Arbaeenia mass gatherings. The attendees included 49.5% (n=6046) females and 50.5% (n=6156) males. The pilgrims' mean age was 33.8 years, with a standard deviation of 15.5 years. Among the pilgrims, 12.6% (n=1534) were aged 10-19 years, 26.1% (n=3180) were aged 20-29 years, 21.8% (n=2662) were aged 30-39 years, and 18.3% (n=2230) were aged 40-49 years. These individuals sought medical assistance at 10 healthcare facilities over 10 days.

Table 1 shows that the majority of the pilgrims, accounting for 77.4% (n=9444), were between the ages of 20 and 59. More than half of the pilgrims were foreigners, accounting for 58.1% (n=7092). Of the foreign pilgrims, 41.9% (n=5110) originated from Iraq (Table 1). Notably, 97% (n=6879), of the foreign pilgrims were from Iran, followed by 1.06% (n=75) from Kuwait.

**Table 1:** Demographic characteristics of pilgrims (n=12202) who attended 10 health clinics during the Arbaeenia mass gathering in 2023 Number of pilgrims (Percentage)

Variables	Category	N (%)
Gender	Male	6156 (50.5)
	Female	6046 (49.5)
Country	Iraq	5110 (41.9)
	Foreign	7092 (58.1)
Age groups	<1	106 (0.9)
	1-9y	422 (3.5)
	10-19y	1534 (12.6)
	20-29y	3180 (26.1)
	30-39y	2662 (21.8)
	40-49y	2230 (18.3)
	50-59y	1372 (11.2)
	60+y	696 (5.7)
Educational level	Child	285 (2.3)
	Illiterate	971 (8)
	Read & write	5126 (42)
	Primary school	1156 (9.5)
	Secondary school	1908 (15.6)
	University	2078 (17)
	Postgraduate	678 (5.6)

There were also smaller proportions of pilgrims from India, Pakistan, and Bahrain, and 25 from seven other countries. Among the 5110 recorded Iraqis, the majority 85.65% (n=4377) came from the Najaf, Basra, and Al Diwaniyah governorates as shown in Table 1.

**Figure 1:**
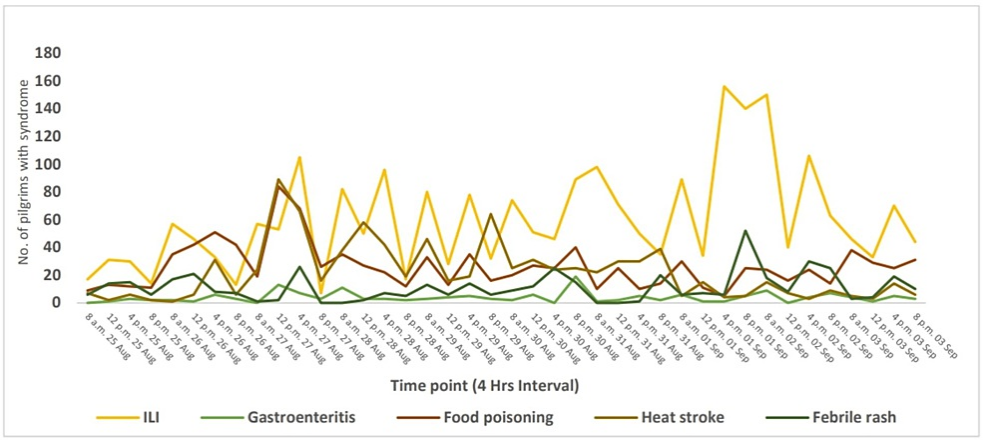
Time trend of the counts of five common syndromes among the time points during Arbaeenia mass gatherings, from August 25, 2023, to September 3, 2023 (n=4938) ILI: influenza like-illness (4 hours interval)

Concerning the temporal trends of the five common syndromes at the time points, we summarize the counts of the five included syndromes from the 1st to 40th points of time; each point of time represents four hours of work, 16 hours in total per day in the health clinic on the road of pilgrims, which means that for each day, we have four points of time. Figure 1 shows that the run chart of the time points-data counts from the first to 40 shows that the points of time data are counts of syndromes for each point of time. The epidemic peak period for influenza-like illness (ILI) syndrome began at 4 p.m. on September 1, 2023. On the other hand, all the remaining syndromes were distributed by their counts as well.

Outbreak detection algorithms

In addition to reporting the time trends of the numbers of syndromes (classified syndromes) in the remainder of this section, using the outbreak detection algorithms numbers of syndromes reported by applied outbreak detection algorithms, including moving average (MA), cumulative sum (CUSUM), and exponentially weighted moving average (EWMA).

Moving Average Algorithm

**Figure 2:**
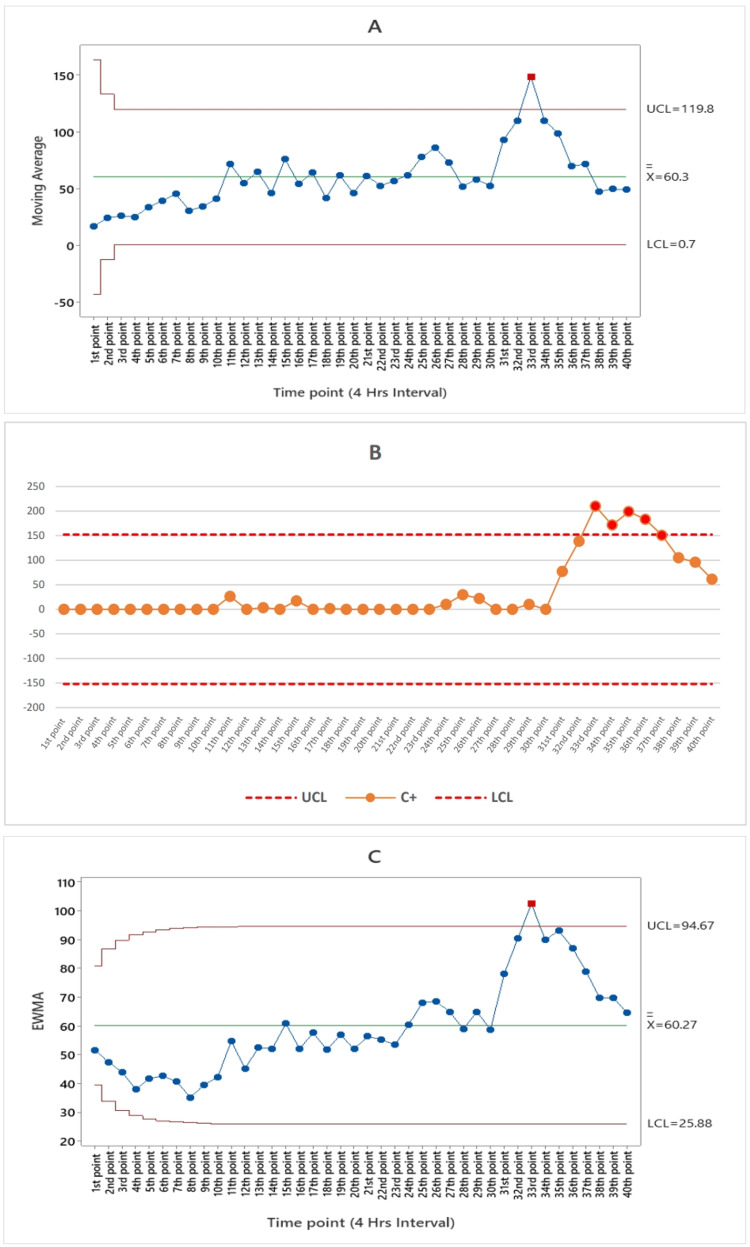
(A) Moving average (MA), (B) the cumulative cum (CUSUM), and (C) exponentially weighted moving average (EWMA) algorithm charts by the numbers of influenza-like illness (ILI) syndrome subfigures from the 1st point of time represented August 25 until the 40th point of time which represents the end of September 3 in 2023 UCL: upper control limit, LCL: lower control limit, C+: CUSUM value (4 hours interval)

The moving average algorithms for ILI syndromes are depicted in Figure 2A, illustrating the occurrence of a single point alarm at the 33rd time point, corresponding to 8 a.m. on September 2, 2023.

**Figure 3:**
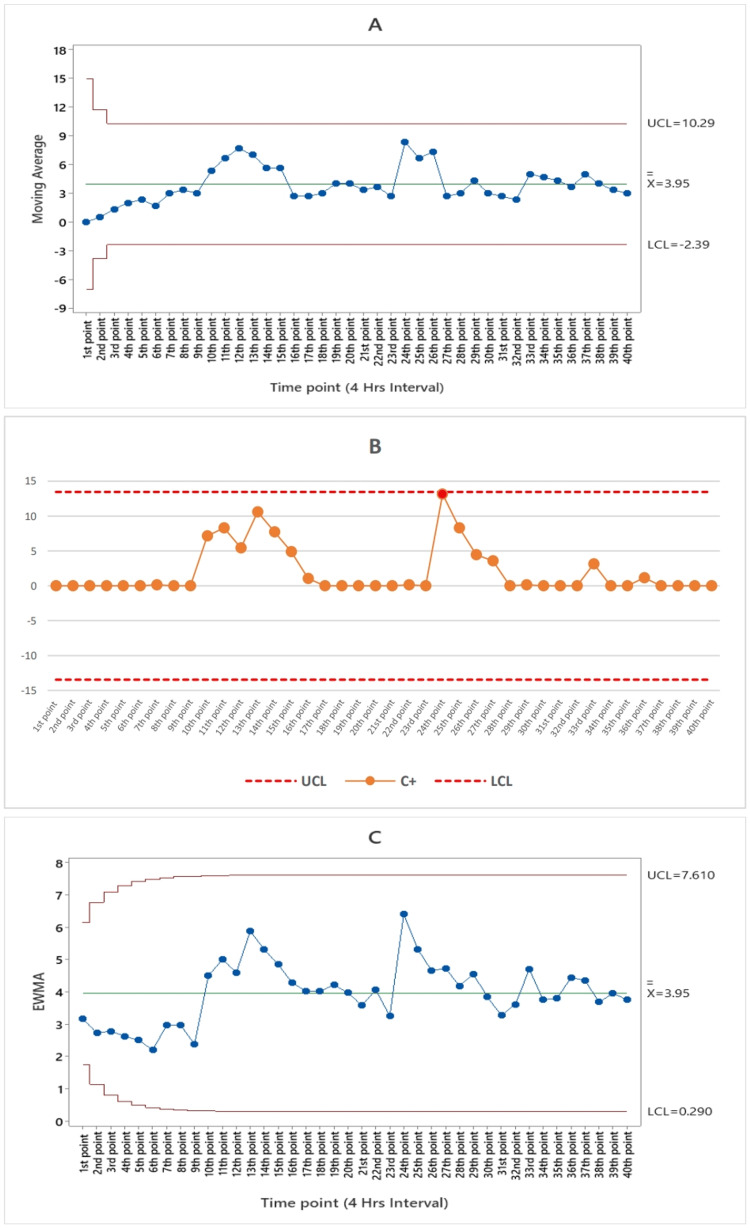
(A) Moving average (MA), (B) the cumulative cum (CUSUM), and (C) exponentially weighted moving average (EWMA) algorithm charts by the numbers of gastroenteritis syndrome subfigures from the 1st point of time represented August 25 until the 40th point of time which represents the end of September 3 in 2023 UCL: upper control limit, LCL: lower control limit, C+: CUSUM value (4 hours interval)

Figure 3A displays the moving average algorithm for gastroenteritis syndrome, revealing that none of the data points exceeded the control limits throughout the 40-point study period.

**Figure 4:**
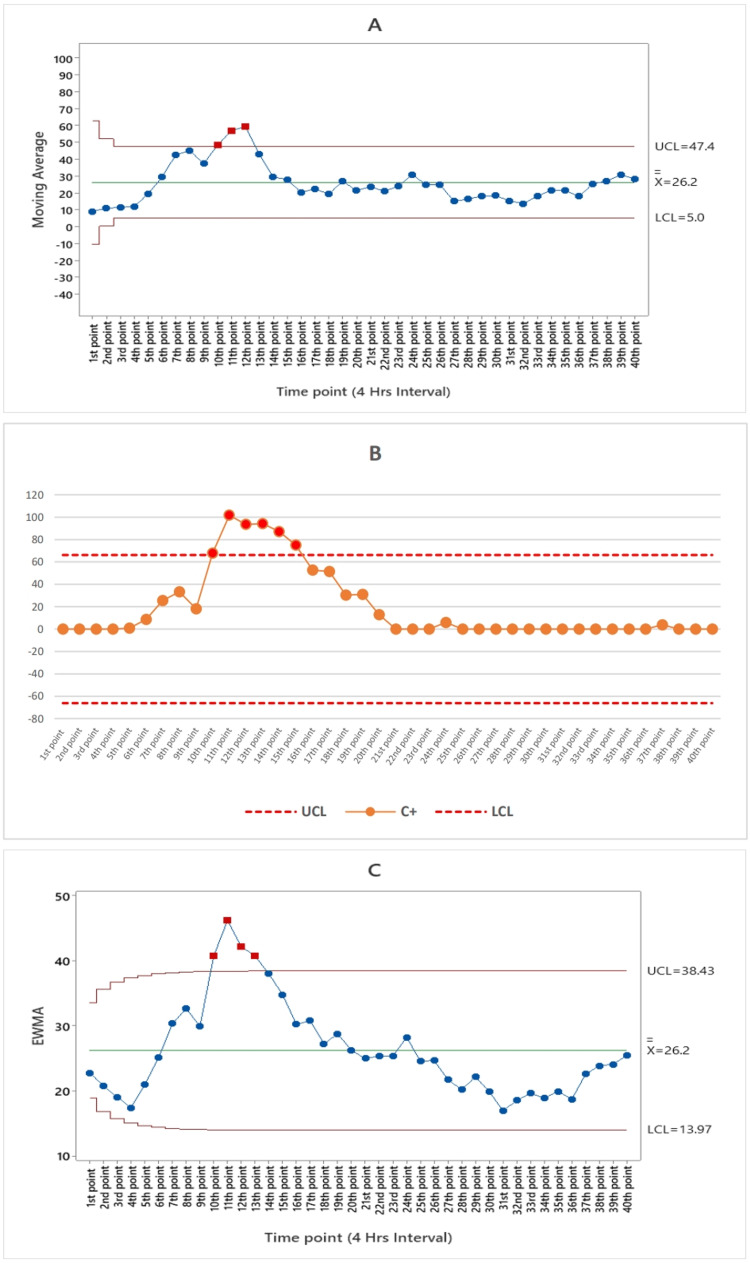
(A) Moving average (MA), (B) the cumulative cum (CUSUM), and (C) exponentially weighted moving average (EWMA) algorithm charts by the numbers of food poisoning syndrome subfigures from the 1st point of time represented August 25 until the 40th point of time which represents the end of September 3 in 2023 UCL: upper control limit, LCL: lower control limit, C+: CUSUM value (4 hours interval)

The moving average algorithm for food poisoning syndrome is presented in Figure 4A, indicating the presence of three alarm points occurring between the 10th and 12th time points, aligning with the time frame from 12 p.m. to 8 p.m. on August 27, 2023.

**Figure 5:**
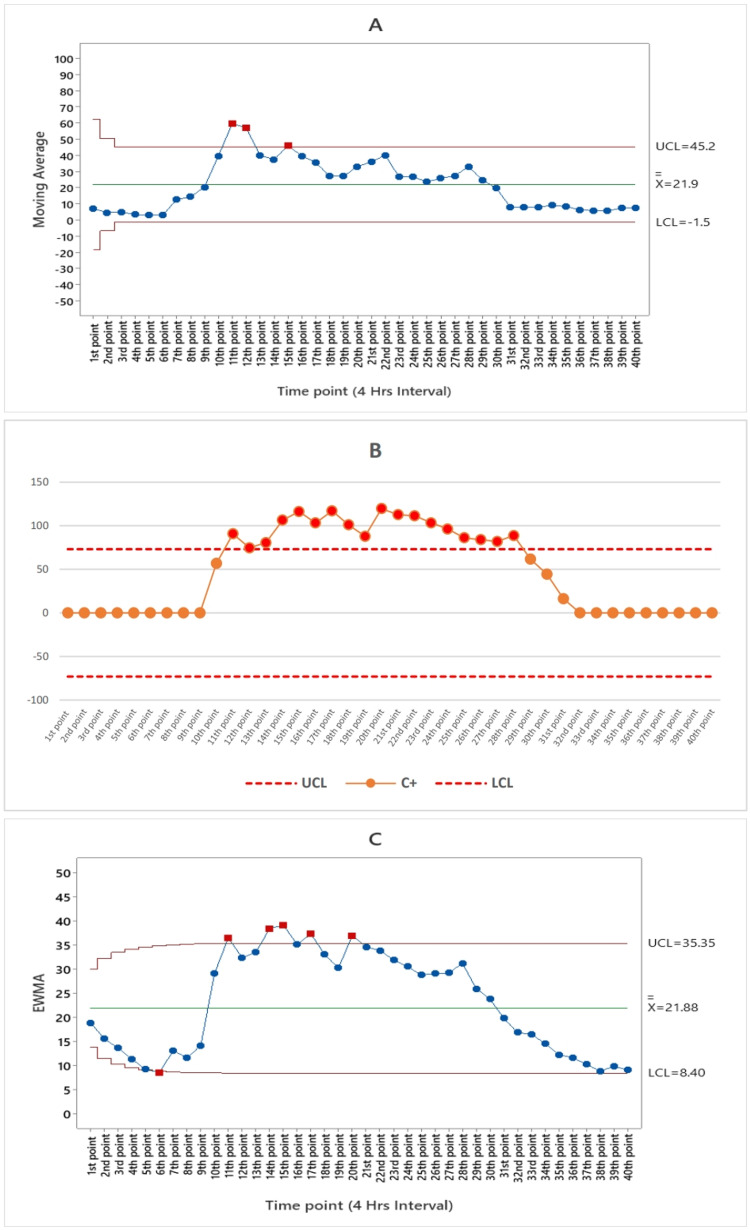
(A) Moving average (MA), (B) the cumulative cum (CUSUM), and (C) exponentially weighted moving average (EWMA) algorithm charts by the numbers of heatstroke syndrome subfigures from the 1st point of time represented August 25 until the 40th point of time which represents the end of September 3 in 2023 UCL: upper control limit, LCL: lower control limit, C+: CUSUM value (4 hours interval)

The moving average algorithm for heatstroke syndrome, as depicted in Figure 5A, illustrates that there are three instances of alarm at the 11th, 12th, and 15th time points, which correspond to 4 p.m., 8 p.m. on August 27, 2023, and 4 p.m. on August 28, 2023.

**Figure 6:**
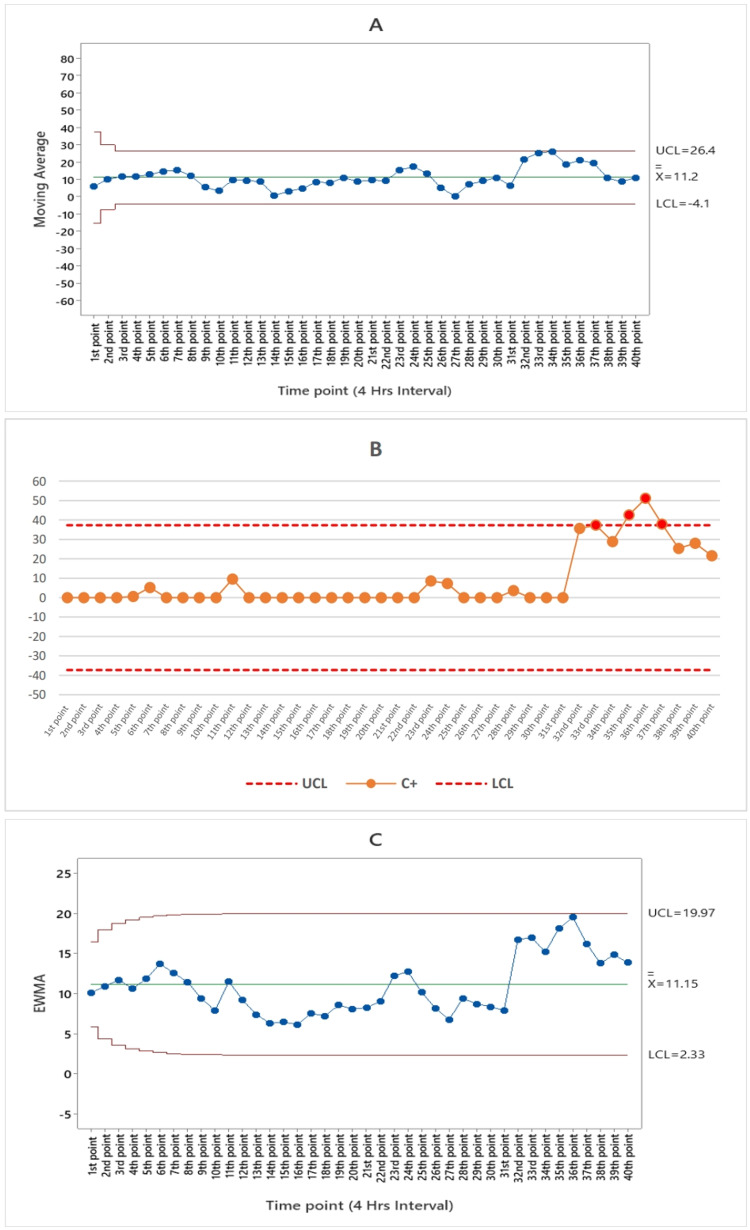
(A) Moving average (MA), (B) the cumulative cum (CUSUM), and (C) exponentially weighted moving average (EWMA) algorithm charts by the numbers of febrile rash syndrome subfigures from the 1st point of time represented August 25 until the 40th point of time which represents the end of September 3 in 2023 UCL: upper control limit, LCL: lower control limit, C+: CUSUM value (4 hours interval)

In Figure 6A, the moving average (MA) algorithm for febrile rash syndrome reveals no alarm occurrence throughout the time course, indicating that febrile rash is in the growth phase.

Cumulative Sum (CUSUM) Algorithm

According to the CUSUM algorithm, there were a total of five alarms for ILI syndrome occurring between the 33rd and 37th time points. These alarms are consistent with the period from 8 a.m. on September 2, 2023, to 8 a.m. on September 3, 2023, as depicted in Figure 2B. Figure 3B illustrates that the cumulative cum algorithm for gastroenteritis syndrome detected a single alarm at the 24th time point, which aligns with 8 p.m. on August 30, 2023. As indicated in Figure 4B, the cumulative sum algorithm for food poisoning syndrome identified six alarms between the 10th and 15th time points. These alarms correspond to the period from noon on August 27, 2023, until 4 p.m. on August 28, 2023. Figure 5B shows that the CUSUM algorithm for the number of heatstroke syndrome cases yielded 18 alarm points that occurred between 11 and 28, which is consistent with the data from 4 p.m. on August 27, 2023, until 8 p.m. on August 31, 2023. Figure 6B shows the results of the CUSUM algorithm for the number of febrile rash syndrome events, which revealed four alarm points that occurred at 33, 35, and 37, which is consistent with the results at 8 a.m. on September 2, 2023, 4 p.m. on September 2, 2023, and 8 a.m. on September 3, 2023.

Exponentially Weighted Moving Average (EWMA) Algorithm

The EWMA algorithm is also shown in Figure 2C; according to the EWMA algorithm for the number of ILI syndrome cases, there is one point alarm that occurred at the 33rd time point at 8 a.m. on September 2, 2023. According to Figure 3C, for the EWMA algorithm for the number of patients with gastroenteritis syndrome, no point alarms occurred during the study period (40 points in time). According to the EWMA algorithm for the number of food poisoning syndrome cases, four-alarm points occurred from the 10th to the 13th, which is consistent with the date noon of August 27, 2023, until the morning of August 28, 2023, as shown in Figure 4C. The exponentially weighted moving average algorithm for the number of heatstroke syndrome events (Figure 5C) indicates that five-alarm points occurred on the 11th, 14th, 15th, 17th, and 20th, which are consistent with the dates of 4 p.m. August 27, 2023, noon to 4 p.m. August 28, 2023, and morning until 8 p.m. August 29, 2023. The exponentially weighted moving average algorithm for the number of febrile rash syndrome patients did not alarm during the study period, as shown in Figure 6C.

## Discussion

The purpose of the current study was to examine the implementation and efficacy of outbreak detection algorithms for syndromic surveillance during mass gatherings in Iraq. Within 10 days of the large assembly, 12202 pilgrims visited the 10 health clinics; 40.5% (n=4938) of them had at least one syndrome. Among the patients with this syndrome, ILI syndrome accounted for 48.8% (n=2411), food poisoning syndrome for 21.2% (n=1048), heatstroke syndrome for 17.7% (n=875), febrile rash syndrome for 9.0% (n=446), and gastroenteritis syndrome for 3.2% (n=158).

Mass gatherings create favorable conditions for infectious disease transmission. This study revealed that more than two-fifths (40.5%) of patients presented with acute symptoms or syndromes. Other studies have previously emphasized an increased risk of infectious disease outbreaks during mass gatherings [7,26]. 

Since enhancing public health surveillance and deploying outbreak detection algorithms for studied syndromes during Arbaeenia mass gatherings in Iraq cannot detect all outbreaks effectively by using one algorithm, using different types of outbreak detection algorithms for syndromes provides useful and important information regarding the strengths and weaknesses of the applied algorithm. This study investigated how these algorithms contribute to improved public health surveillance efforts, particularly in identifying and managing potential outbreaks or health concerns during mass gatherings in Iraq.

Our findings indicate that analyzing real-time data on ILI, gastroenteritis, food poisoning, heatstroke, and febrile rash syndromes or symptoms during Arbaeenia mass gatherings can be valuable for identifying potential outbreaks, particularly those that emerge rapidly. This approach complements the traditional surveillance system which relies on laboratory confirmation. By comparing various predictors, we discovered alarm signals in the data collected from the Arbaeenia mass gatherings in Iraq. Notably, the analysis of health clinic visits along the roads of Najaf and Karbala revealed multiple waves of alarm for different syndromes under study, with ILI and heatstroke syndromes exhibiting a greater frequency of alarm than the other syndromes examined compared to other studies involving communicable disease outbreaks of various infectious diseases, which have been reported repeatedly during and following Hajj [27].

Strengths and limitations

Public health officials and the government will remain informed, vigilant, and active after monitoring the variation with control charts. This study highlights the weak aspects of public health surveillance and mass gatherings. This approach will facilitate a better understanding of the phases of outbreak detection, hence, allowing for better and proper handling of any outbreaks during mass gatherings in Arbaeenia.

This study is limited to monitoring the variation in the number of syndromes followed during this large religious event. No comparison is made among the control charts. The efficiency of control charts can be compared based on the speed of the charts in detecting alarming deaths due to syndromes. The accuracy of the reported data limits the findings. The more accurate the reported data are, the better the findings.

This article describes the use of CUSUM to detect aberrant syndromes. Four aberrant syndromes were detected during Arbaeenia mass gatherings with different alarms, including ILI, food poisoning, heatstroke, and febrile rash as shown in Figures 2, 4, 5, 6 respectively. In addition to detecting aberrant syndromes, the cumulative cum method can also be used as a diagnostic tool to identify compromised items. Lee and Lewis adopted the CUSUM algorithm, which is preferable for detecting items that might be exposed during continuous testing for real-time data [28].

A specific algorithm like the EWMA can detect large shifts for all studied syndromes effectively. Accordingly, it is necessary to use this algorithm throughout mass gatherings and in syndromic surveillance systems to provide useful and important information regarding the strengths and weaknesses of the applied algorithm. The EWMA is often superior to CUSUM for large shifts. A similar study was performed by Steiner et al. to determine the onset and end of seasonal influenza outbreaks [29]. They concluded that the EWMA could detect the start date of a seasonal influenza outbreak within one or two weeks after the actual start date of the influenza outbreak.

According to the findings of numerous researchers, it is widely acknowledged that no single algorithm exists that can effectively handle a diverse array of outbreaks occurring in various circumstances [23,30]. While we attempted to address this issue by employing a moving average to preprocess daily counts of syndromes, thereby taking into account the assumption of normality, it is possible that the unsatisfactory performance of EWMA algorithms can be attributed, at least in part, to a violation of the algorithm's underlying assumption.

Recommendations

Our recommendations for future research are to conduct longitudinal studies and track syndromic surveillance data over time during mass gatherings to understand disease outbreak dynamics better, compare different surveillance algorithms to enhance outbreak detection and monitoring, explore combining syndromic surveillance data with other health information sources like electronic records or social media for better early detection, and investigate specific disease patterns during mass gatherings to develop targeted prevention strategies.

## Conclusions

In conclusion, the findings of this study underscore the immense strain placed on local healthcare systems during the mass gathering of Arbaeenia in Iraq, with a substantial number of pilgrims seeking medical care for various syndromes. The analysis revealed that over 40% of patients presented with acute symptoms; by analyzing real-time data on syndromes such as influenza-like illness (ILI), gastroenteritis, food poisoning, heatstroke, and febrile rash, authorities gained critical insights into emerging health threats. The study revealed multiple alarm signals, with ILI and heatstroke syndromes exhibiting higher frequencies, indicating the effectiveness of these surveillance methods. This study contributes to improving public health surveillance efforts, and comparisons among control charts could provide insights into their relative efficiency in detecting alarming syndromes. Additionally, the accuracy of the reported data remains crucial for the reliability of the findings.

The use of the cumulative sum (CUSUM) method proved effective in detecting aberrant syndromes, offering a diagnostic tool for identifying compromised items. However, specific algorithms such as the exponentially weighted moving average (EWMA) may be more effective in detecting large shifts for all studied syndromes, indicating the need for a comprehensive approach in syndromic surveillance systems. This study underscores the importance of robust public health surveillance systems, particularly during mass gatherings, to promptly detect and respond to emerging health threats. By leveraging advanced algorithms and real-time data analysis, authorities can enhance their preparedness and response capabilities, ultimately safeguarding public health during such events.

## Appendices

**Table 2:** Information sheet

No.	Name of variable	Variable type		Definition of variable	Measurement tool and method
Part 1_Demographic characteristics					
1	Date	Qualitative variable (nominal)	Background variable	It can be useful in identifying an outbreak according to the time trend	Questionnaire -Calendar
2	Time (hours)	Quantitative variable (discrete)	Background variable		Questionnaire -Calendar
3	Age (years)	Quantitative variable (continuous)	Background variable	-Based on date of birth -Can be useful in identifying outbreaks and alerts, and in monitoring the progression of an epidemic that affects specific age groups	Questionnaire
4	Gender	Qualitative variable (nominal)	Background variable	-Gender of participants -Can be a useful source of information as it can provide additional information on the makeup of an outbreak	Male (1) Female (0)
5	Educational level	Qualitative variable (ordinal)	Independent variable	-Levels of education of participants in the current study -Useful for identifying which level of education can be affected	Illiterate (1) Read & Write (2) Primary School (3) Secondary School (4) University (5) Postgraduate (6)
6	Country	Qualitative variable (nominal)	Independent variable	-The original country of participants in the current study -This part is important to detect if there are any new imported cases regarding communicable diseases	Iraq (1) Foreign (0)
Part 2_Acute or infectious conditions or syndromes					
7	Fever of ≥ 38 C°	Qualitative variable (nominal)	Independent variable	These signs and symptoms (syndromes) are related to the public health threats like influenza, respiratory pathogens, chemical incidents, etc.	No (0) Yes (1)
8	Cough	Qualitative variable (nominal)	Independent variable		No (0) Yes (1)
9	Sore throat	Qualitative variable (nominal)	Independent variable		No (0) Yes (1)
10	Shortness of breath	Qualitative variable (nominal)	Independent variable		No (0) Yes (1)
11	Nausea	Qualitative variable (nominal)	Independent variable	According to these syndromes, we can indicate foodborne illness	No (0) Yes (1)
12	Vomiting	Qualitative variable (nominal)	Independent variable		No (0) Yes (1)
13	Diarrhea	Qualitative variable (nominal)	Independent variable		No (0) Yes (1)
14	Abdominal pain	Qualitative variable (nominal)	Independent variable		No (0) Yes (1)
15	Acute bloody diarrhea	Qualitative variable (nominal)	Independent variable	According to these syndromes, we can indicate bacterial diseases	No (0) Yes (1)
16	Acute watery diarrhea	Qualitative variable (nominal)	Independent variable	Can be a useful source of information as it can provide additional information on viral, bacterial, or parasitic outbreaks during mass gatherings	No (0) Yes (1)
17	Skin rash	Qualitative variable (nominal)	Independent variable	According to these syndromes, we can indicate alarm for some communicable conditions like measles, rubella, chickenpox, etc.	No (0) Yes (1)
18	Confusion	Qualitative variable (nominal)	Independent variable	Health indicator for heatstroke	No (0) Yes (1)
19	Coma	Qualitative variable (nominal)	Independent variable		No (0) Yes (1)
20	Profuse sweating	Qualitative variable (nominal)	Independent variable		No (0) Yes (1)
21	Headache	Qualitative variable (nominal)	Independent variable		No (0) Yes (1)
